# Supplementary material for: Identification of Phage Receptor-Binding Protein Sequences with Hidden Markov Models and an Extreme Gradient Boosting Classifier
Source: Viruses. 2022 Jun 17;14(6):1329. doi: 10.3390/v14061329 (PMC9230537; doi:10.3390/v14061329)
Supplement: Supplementary file 1 [file viruses-14-01329-s001.zip › viruses-1729238-supplementary.pdf]

Identification of Phage Receptor-Binding Protein Sequences with Hidden Markov Models and an Extreme Gradient Boosting Classifier

Dimitri Boeckaerts [1,2], Michiel Stock [2,3], Bernard De Baets [2] and Yves Briers [1,*]

Supplementary Materials

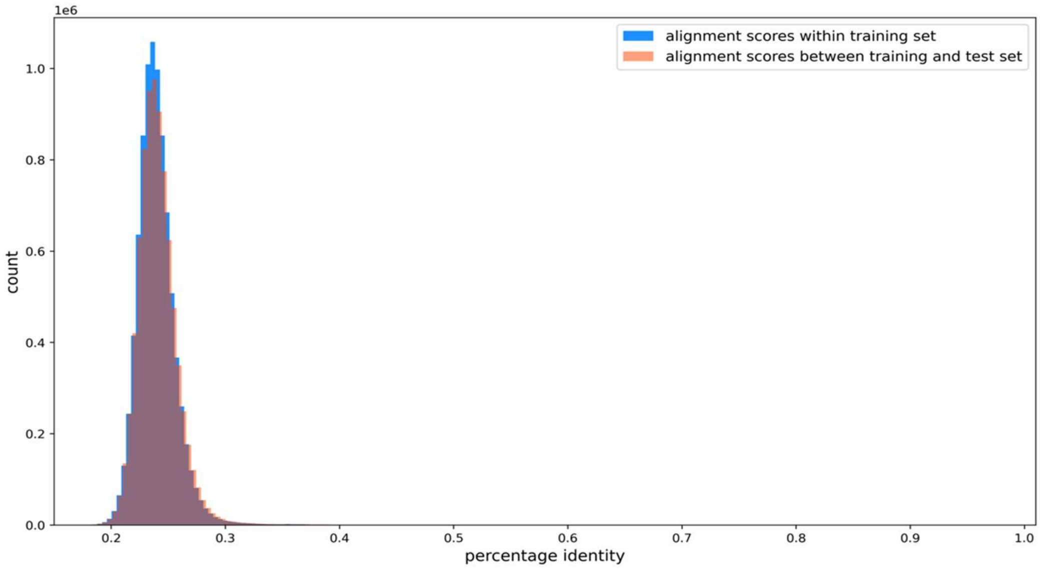

Figure S1: Histograms of pairwise local alignment identity percentages between each of the RBP sequences in the training set and each of the RBP sequences in the test set (orange), as well as for all the RBP sequences within the training set (blue). The pairwise alignments were computed with the BioJulia package within Julia. The histograms indicate that the similarity within the training data is not different from the similarity between the training and the test data.
